# Optimized automated radiosynthesis of ^18^F-JNJ64413739 for purinergic ion channel receptor 7 (P2X7R) imaging in osteoporotic model rats

**DOI:** 10.3389/fphar.2024.1517127

**Published:** 2024-12-12

**Authors:** Yingtong Lu, Yan Cui, Lu Hou, Yuanfang Jiang, Jingjie Shang, Lu Wang, Hao Xu, Weijian Ye, Yang Qiu, Bin Guo

**Affiliations:** ^1^ Department of Nuclear Medicine, The First Affiliated Hospital, Jinan University, Guangzhou, China; ^2^ Traditional Chinese Medicine Department, The First Affiliated Hospital, Jinan University, Guangzhou, China; ^3^ Department of Gynecology, Jiangmen Wuyi Traditional Chinese Medicine Hospital of Jinan University, Jiangmen, Guangdong, China

**Keywords:** P2X7R, osteoporosis, positron emission tomography, isotope labeling, fluorine radioisotopes

## Abstract

**Objective:**

To optimize the automated radiosynthesis of the purinergic ion channel receptor 7 (P2X7R) imaging agent ^18^F-JNJ64413739 and evaluate its potential for brain imaging in osteoporotic model rats.

**Methods:**

A more electron-deficient nitropyridine was employed as the labeling precursor to facilitate the ^18^F-labeling. The radiosynthesis was conducted on an AllinOne synthesis module, and followed by purification via high-performance liquid chromatography (HPLC). The resulting ^18^F-JNJ64413739 was subjected to quality control tests. Small-animal PET/CT imaging studies were performed in sham and osteoporotic model rats.

**Results:**

The optimized automated radiossynthesis of ^18^F-JNJ64413739 was successfully completed in approximately 100 min with non-decay-corrected radiochemical yield of 6.7% ± 3.8% (n = 3), >97% radiochemical purity and >14.3 ± 1.3 GBq/μmol molar activity. The product met all clinical quality requirements. ^18^F-JNJ64413739 PET/CT imaging showed revealed significantly higher radioactivity uptake in various brain regions of the osteoporotic model rats compared to sham control group.

**Conclusion:**

We successfully optimized the automated radiosynthesis of ^18^F-JNJ64413739. The resulting tracer not only met clinical quality requirements but also demonstrated potential for clinical application in the diagnosis of osteoporosis, as evidenced by higher radioactivity uptake in various brain regions of osteoporotic model rats compared to normal controls.

## 1 Introduction

Osteoporosis is primarily a chronic degenerative skeletal disease characterized by reduced bone mass, destruction of bone microstructure, and increased bone fragility, leading to a high susceptibility to fractures among patients (Author anonymous, 1993). The gradual decline in bone mass with age, coupled with fractures stemming from age-related bone loss, represents a main cause of disability and mortality in elderly individuals, thereby exacerbating the global economic burden of osteoporosis (Clynes et al., 2020). Consequently, aging-related osteoporosis, a pressing public health issue, is becoming increasingly prevalent with the aging global population.

Osteoporosis is a complex disease that results from the interaction between genetic and environmental factors (Schultz and Wolf, 2019). Increasing evidence from clinical studies has demonstrated that inflammatory diseases are frequently accompanied by significant local or systemic bone loss, highlighting a strong connection between inflammation and osteoporosis (Chotiyarnwong and McCloskey, 2020; Raterman et al., 2020; Kanazawa et al., 2010; Mazzaferro et al., 2018; Baldini et al., 2005). Notably, early research has shown that women with postmenopausal osteoporosis exhibit elevated levels of serum inflammatory markers and P2X7 receptor (P2X7R) (Xiao et al., 2019; Wang et al., 2018). Further investigations into the role of adenosine triphosphate (ATP) and its associated receptors have indicated their involvement in inflammatory response. P2X7R, an ATP-gated cation-selective channel, plays a crucial role in both inflammation and host defense, and is widely distributed in osteocytes, osteoclasts, and osteoblasts, where it is pivotal to bone metabolism. Activation of P2X7R may alleviate osteoporosis by regulating the balance between osteoblasts and osteoclasts (Dong et al., 2020; Ma et al., 2021). Additionally, functional polymorphisms in the P2X7R gene have been shown to significantly influence osteoporosis (Husted et al., 2013), identifying P2X7R as a promising therapeutic target for inflammatory osteoporosis (Huang et al., 2023). Moreover, literature also suggests that P2X7R in microglia within the central nervous system plays a role in affect the regulation of bone metabolism (Nakazato et al., 2014), indicating a potential regulatory pathway between the brain and bone.

P2X7R is widely distributed throughout the central nervous system, particularly in microglia and astrocytes, where it is activated by ATP and plays a critical role in neuroinflammation (Jimenez-Mateos et al., 2019). Given the importance of P2X7R in these processes, it has become an attractive target for molecular imaging using Positron Emission Tomography (PET). PET imaging, an extensively used technique, is capable of non-invasive and real-time visualization of molecular changes *in vivo* through the use of highly selective probes without inducing pharmacological effects. Among the PET probes targeting P2X7R, the most promising tracer is ^18^F-JNJ64413739, reported by Kolb et al. (2019), which showed the upregulation of P2X7R in a rat model of epilepsy. In 2019, Michel Koole et al. further investigated its radiation dosimetry and target occupancy in three healthy individuals, indicating its potential for clinical imaging of P2X7R (Koole et al., 2019). However, the probe’s remarkably low synthetic yield (3.1% ± 2.0%) (Kolb et al., 2019), likely due to the use of chloropyridine as a labeling precursor, constrains its further clinical applications.

To date, no studies have reported on the use of P2X7R probes for brain imaging in a rat model of osteoporosis. In this study, we aim to employ a more electron-deficient nitropyridine as a labeling precursor to improve the labeling yield through an aromatic nucleophilic substitution reaction. We will also perform small-animal PET/CT imaging on a rat model of osteoporosis to explore the change of P2X7R in this condition.

## 2 Materials and methods

### 2.1 Preparation of radiopharmaceuticals

#### 2.1.1 Radiopharmaceutical reagents and materials

The precursor (S)-(6-methyl-1-(pyrimidin-2-yl)-1,4,6,7-tetrahydro-5H-[1,2,3] triazolo [4,5-c] pyridin-5-yl) (3-nitro-2-(trifluoromethyl)pyridin-4-yl) methanone (Author anonymous, 1993) and the reference standard compound ^19^F-JNJ-64413739 (S)-(3-fluoro-2-(trifluoromethyl)pyridin-4-yl) (6-methyl-1-(pyrimidin-2-yl)-1,4,6,7-tetrahydro-5H-[1,2,3]triazolo [4,5-c]pyridin-5-yl) methanone were made in house. All other chemicals and reagents were obtained from commercial sources and used without any further purification. Solid-phase extraction (SPE) cartridges, including SepPak QMA light (186004540) and SepPak C18 Plus light (WAT023561), were purchased from Waters (Milford, MA, United States). The SPE cartridge SepPak QMA light was pre-conditioned with potassium carbonate (K_2_CO_3_) solution (7.5%, 2.5 mL) and followed by deionized water (10 mL). The C18 Plus light cartridge was re-conditioned with ethanol (EtOH; 5 mL) and followed by sterile water (10 mL). Sterile filter (Millex-GV, 0.22 μM) and vent filter (Millex-FG, 0.20 μM, hydrophobic PTFE) were purchased from Merck-Millipore (Burlington, Massachusetts, United States).

[^18^F]fluoride was generated via the ^18^O (*p*,*n*)^18^F reaction in a 10MeV GE Qilin MINITrace cyclotron using >98% enriched H_2_^18^O (TAIYO NIPPON SANSO Corporation, Tokyo, Japan). Analytical high-performance liquid chromatography (HPLC) was performed using a Shimadzu Essential LC-16 system equipped with an Eckert & Ziegler Mini-Scan/FC radiation detector, with a reverse-phase analytical column (GL Sciences WondaSil C18-WR column, 4.6 mm × 150 mm). Radionuclide purity was tested on an energy disperse spectroscopy (Beijing Sunvic Co., Ltd.).

#### 2.1.2 Synthesis of ^18^F-JNJ-64413739

**FIGURE 1:**
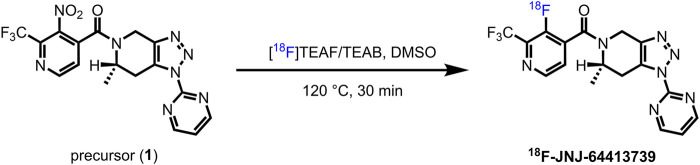
Synthesis route of ^18^F-JNJ6441373.

The synthetic route of ^18^F-JNJ-64413739 was shown in Figure 1. The radiolabeled compound was designed to be synthesized through a S_N_Ar reaction. Utilizing a more electron-deficient nitropyridine precursor, 1. Initially, manual labeling experiments were conducted in small scales. Irradiated [^18^O] water from the cyclotron target (∼74 MBq, 2 mCi) was passed through a pre-conditioned QMA cartridge to trap the [^18^F] fluoride. Subsequently [^18^F] fluoride was eluted with 1 mL of a solution containing the phase transfer catalyst tetraethylammonium bicarbonate (TEAB) in methanol. After elution, the [^18^F]fluoride was dried by azeotropic distillation using N_2_ at 110°C, with two successive additions of dry acetonitrile (2 × 1 ml) to generate a [^18^F] TEAF/TEAB mixture. The precursor 1, dissolved in DMSO, was added to the mixture and heated to 120°C for 30 min, followed by quenching with water (2 ml). The crude product was analyzed by analytical HPLC with a mobile phase comprised CH_3_CN/H_2_O (*v*/*v*, 40/60) at a flow rate of 1 mL/min.

**FIGURE 2:**
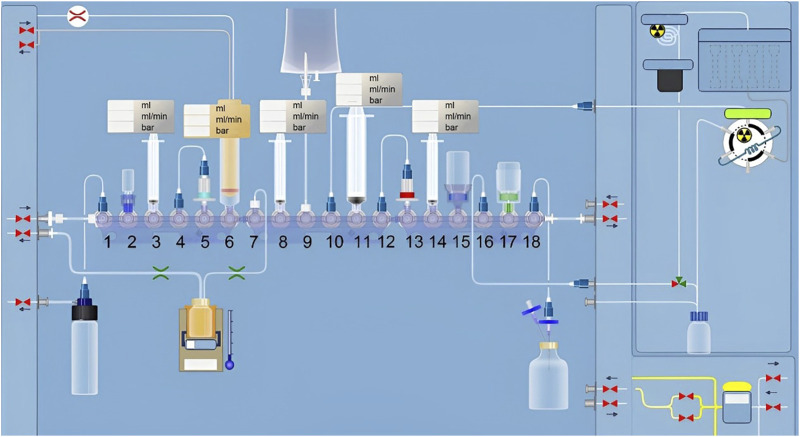
Flowchart of automated synthesis of ^18^F-JNJ64413739 using the All in One module.

Figure 2 presents a schematic diagram of Trasis AllinOne radiosynthesis module used for the synthesis of ^18^F-JNJ-64413739. The automated synthesis involved the following steps:

(1) Azeotropic evaporation and preparation of [^18^F]TEAF. [^18^F]fluoride entered the module through position 6 and was trapped by a Sep-Pak QMA cartridge. It was then eluted with TEAB solution (2 mg TEAB in 1 ml methanol) pre-loaded in a syringe at position 3. The eluent was transferred into the reaction vial and heated at 85°C for 5 min. Acetonitrile (1 ml) on the bottle at position 2 was added into the reaction vial, and the mixture was heated at 110°C for 7 min to obtain [^18^F] TEAF/TEAB.
(2) ^18^F-labeling. The precursor (1 mg of compound 1 dissolved in 0.6 ml of DMSO) in the syringe at position 8 was added to the reaction vial. The vial was sealed and heated at 120°C for 20 min.
(3) Purification. The reaction mixture was injected onto a semi-preparative column (OSAKA SODA, CAPCELL PAK C18 UG80 S5, 5 μm, 10 mm × 250 mm), and eluted with CH_3_CN/H_2_O (*v*/*v*, 35/65) containing 0.1% Et_3_N, with a flow rate of 3.0 mL/min and UV detection at 254 nm.
(4) Formulation. The product fraction was collected in a receiving bottle containing 30 ml of water at position 8. It was then captured by the C18 cartridge at position 13. After rinsing with 10 ml of water, the product was eluted from the cartridge with 1 ml of ethanol pre-loaded in the syringe at position 14, then diluted with water (9 ml, containing 0.5% sodium ascorbate) at position 17. This solution was then passed through a 0.22 μm membrane filterand collected in a sterile vial to ensure the sterility of the final product.

#### 2.1.3 Quality control

The appearance of the product was assessed by visual inspection through lead glass to estimate the color and clarity. A pH-indicator strip was used to measure the pH of the product solution. The radionuclidic identity was confirmed by estimating the half-life of the radionuclid. The identity, radiochemical purity, radiochemical stability, and chemical purity of the product were determined by an analytical HPLC system equipped with a GL Sciences WondaSil C18-WR column (4.6 mm × 150 mm). The mobile phase consisted of CH_3_CN/H_2_O (*v*/*v*, 40/60) with the flow rate of 1 mL/min and UV detection at 254 nm.

### 2.2 Animals and treatment

Six female Sprague–Dawley (SD) rats, 12 weeks old, were purchased from the Beijing Weitong Lihua Biotechnology Co., Ltd., (SCXK [Yue] 2022–0063). Temperature (23°C ± 2°C), relative humidity (45%–50%) and (12 h light/dark cycle) were maintained in the feeding environment. The rats had free access to food and water and were acclimatized to the laboratory environment for 1 week before surgery. The animal experiment was ethically approved by the Laboratory Animal Ethics Committee in the Institute of Jinan University (IACUC-20230307-01). The rats divided into two groups: a sham control group (Sham; sham surgery with phosphate-buffered saline tail vein injections) and an ovariectomy control group (OVX; ovariectomy surgery with phosphate-buffered saline treatment), each consisting of three rats. The specific animal modeling methods were refer to previous literature (Liu et al., 2018).

### 2.3 Small-animal PET/CT imaging

Each rat was anesthetized by 2.5%–3% isoflurane inhalation, and an intravenous catheter was inserted into the tail vein. The radiotracer ^18^F-JNJ-64413739 (*ca*. 15–20 MBq in 150–200 μL) was injected via the pre-installed catheter, and 45 min later, brain PET/CT images were acquired over 10 min. PET/CT imaging experiments were performed on an IRIS small-animal PET/CT imaging system (inviscan SAS, Strasbourg, France).

The PET data were reconstructed with three-dimensional ordered-subset expectation-maximizaton (3D-OSEM) algorithm with a Monte-Carlo based accurate detector model. The resulting PET images were then summed and co-registered with the rat brain MRI template using PMOD software (version 4.1). The radioactivity was decay-corrected to the injection time and expressed as the standardized uptake value (SUV) which was normalized to the injected radioactivity and body weight. The High Resolution CT (HRCT) data were acquired with tube voltage at 80 kV, a single exposure time was 56 ms, a field of view (FOV) of 9 cm, a scanning resolution of 60 µm.

### 2.4 Bone mineral density and Micro-CT

The bone mineral density (BMD) of the lumbar vertebrae and bilateral femurs was analyzed by dual-energy X-ray absorptiometry (DXA) (General Electric Company, Healthcare), employing specific software designed for assessing bone density in small animals. The results are analyzed by lunar_iDXA software and presented in g/cm^2^.

The femurs were separated and carefully dissected free of soft tissues. The bone samples were vertically fixed in the sample fixator along the long axis, and imaging was performed using a Hiscan XM Micro CT (Suzhou Hiscan Information Technology Co., Ltd. Suzhou, Jiangsu Province, China). The scanning conditions were set to 80 kV and 100 μA, with a single exposure time was 50 ms, a scanning resolution of 25 μm, and a scanning angle interval of 0.5°. Reconstruction and analysis were conducted with Hiscan Reconstruct software and Hiscan Analyzer software (Version 3.0, Suzhou Hiscan Information Technology Co., Ltd., Jiangsu Province, China). Subsequently, the bone volume/tissue volume (BV/TV), trabecular number (Tb.N), trabecular thickness (Tb.Th), and trabecular separation (Th.Sp) data were collected.

### 2.5 Hematoxylin and eosin staining

Samples taken from the femur were fixed with 4% paraformaldehyde, decalcified with 18% EDTA, dehydrated, and embedded with paraffin. Subsequently, 5 μm thick sections were placed on glass slides and stained with hematoxylin and eosin (H&E). The 10 μm thick sections were prepared, stained, counterstained, dehydrated, hyalinized, and mounted. The antibody dilution for IHC was 1:100. All images were captured using a microscope (Zeiss AXIO).

### 2.6 Statistical analysis

Data were presented as mean ± standard deviation (SD). Statistical analysis were performed using SPSS 22.0 software. All experiments were repeated at least 3 independent times, and representative results are shown. A value of *p* < 0.05 was considered statistically significant.

## 3 Results

### 3.1 Radio synthesis

**FIGURE 3:**
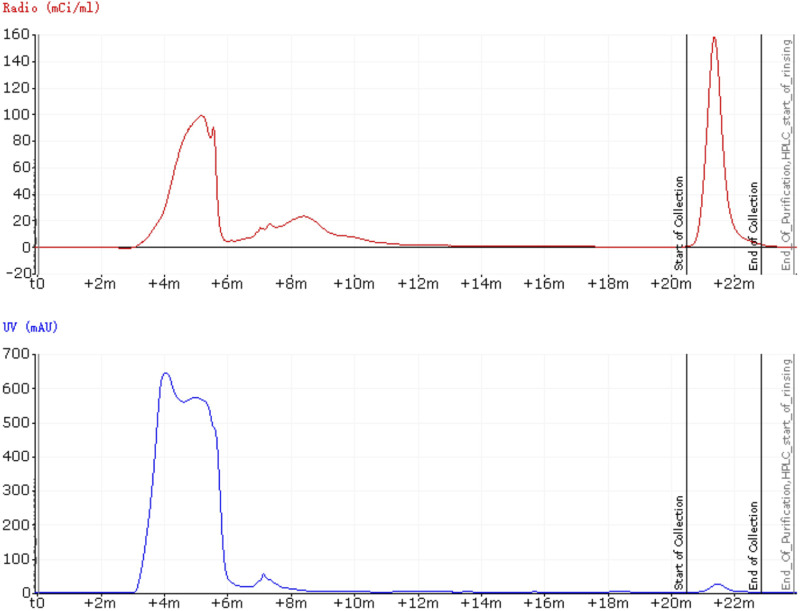
Semi-preparative HPLC spectrum of ^18^F-JNJ64413739.

Before developing an automated radiosynthesis of ^18^F-JNJ-64413739, initial manual labeling experiments were conducted to screen various reaction conditions. Starting with 1 mg of precursor (1), ^18^F-JNJ-64413739 was synthesized by eluting the trapped [^18^F]fluoride form QMA using a solution of 2 mg TEAB dissolved in 1 ml methanol, followed by heating at 120°C for 30 min. The manual labeling experiments showed that the precursor 1 could smoothly transform into ^18^F-JNJ-64413739. With the success of these initial small-scale manual experiments, the automated synthesis was then carried out. The process was successfully completed, and the retention time of ^18^F-JNJ-64413739 on the semi-preparative HPLC was observed at 21.5 min (Figure 3). The entire automated synthesis process, from the end of cyclotron bombardment to obtaining the ^18^F-JNJ-64413739 product, took approximately 100 min. Using the optimized process, three consecutive batches were produced, achieving non-corrected synthesis yields (n. d. c. RCY) of 6.7% ± 3.8%, ranging from 3% to 12%, with specific activities greater than 14.3 ± 1.3 GBq/μmol (n = 3).

### 3.2 Quality control

**FIGURE 4:**
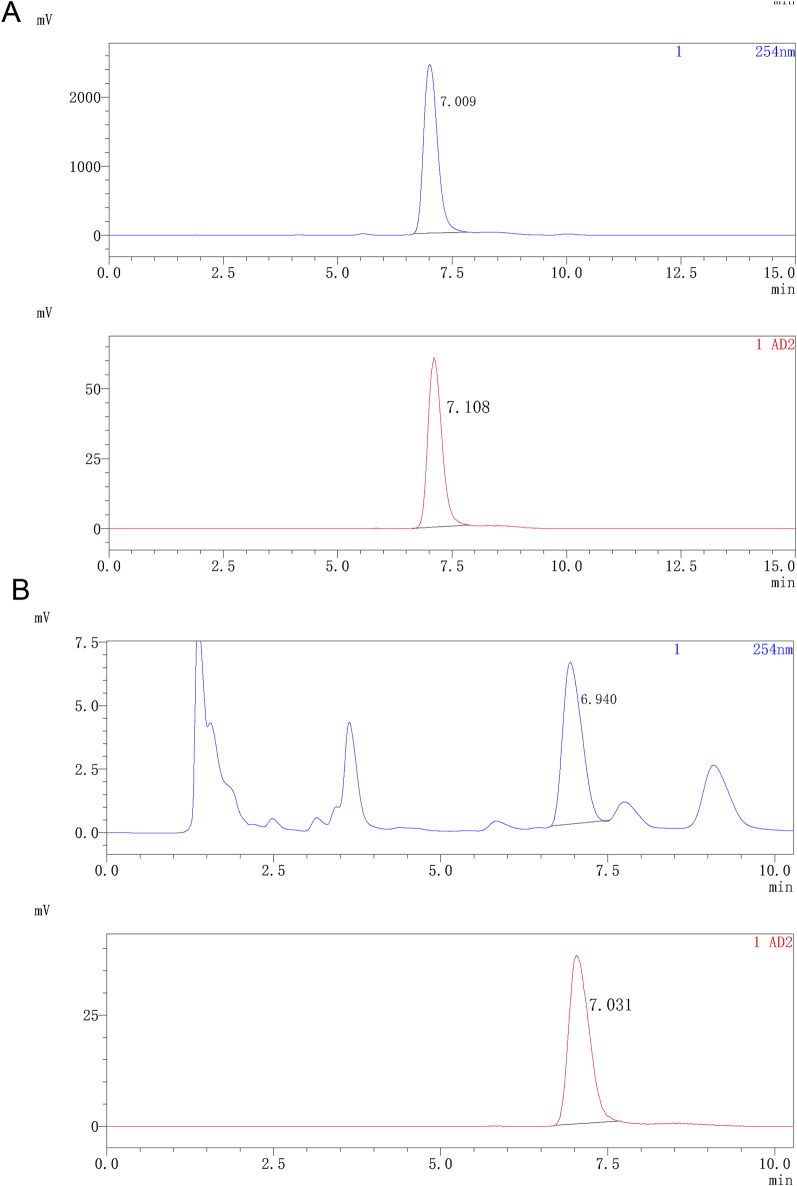
Analytical HPLC spectra of ^18^F-JNJ64413739. **(A)** HPLC spectrum of the product co-injected with the standard reference; **(B)** HPLC spectrum of the product after 5 h. The upper spectrum shows UV signal, while the lower spectrum shows radioactivity signal.

Indicating compatibility with intravenous injection, the obtained ^18^F-JNJ-64413739 solution was colorless and transparent without any suspended particles, with a pH of 7.0 and a radionuclide half-life of 110 min. As shown in Figure 4, a co-injection of the tracer and the standard with analytical HPLC chromatogram showed that the UV absorption peak and the radioactive peak appeared at 7.009 and 7.108 min, respectively, indicating that the synthesized radiotracer was confirmed to be ^18^F-JNJ-64413739. Throughout the validation process, the nitropyridine precursor, compound 1, was below the limit of detection in any of the batches. The radiochemical purity of the product was greater than 97% and remained over 95% after 5 h at room temperature.

### 3.3 Imaging results

**FIGURE 5:**
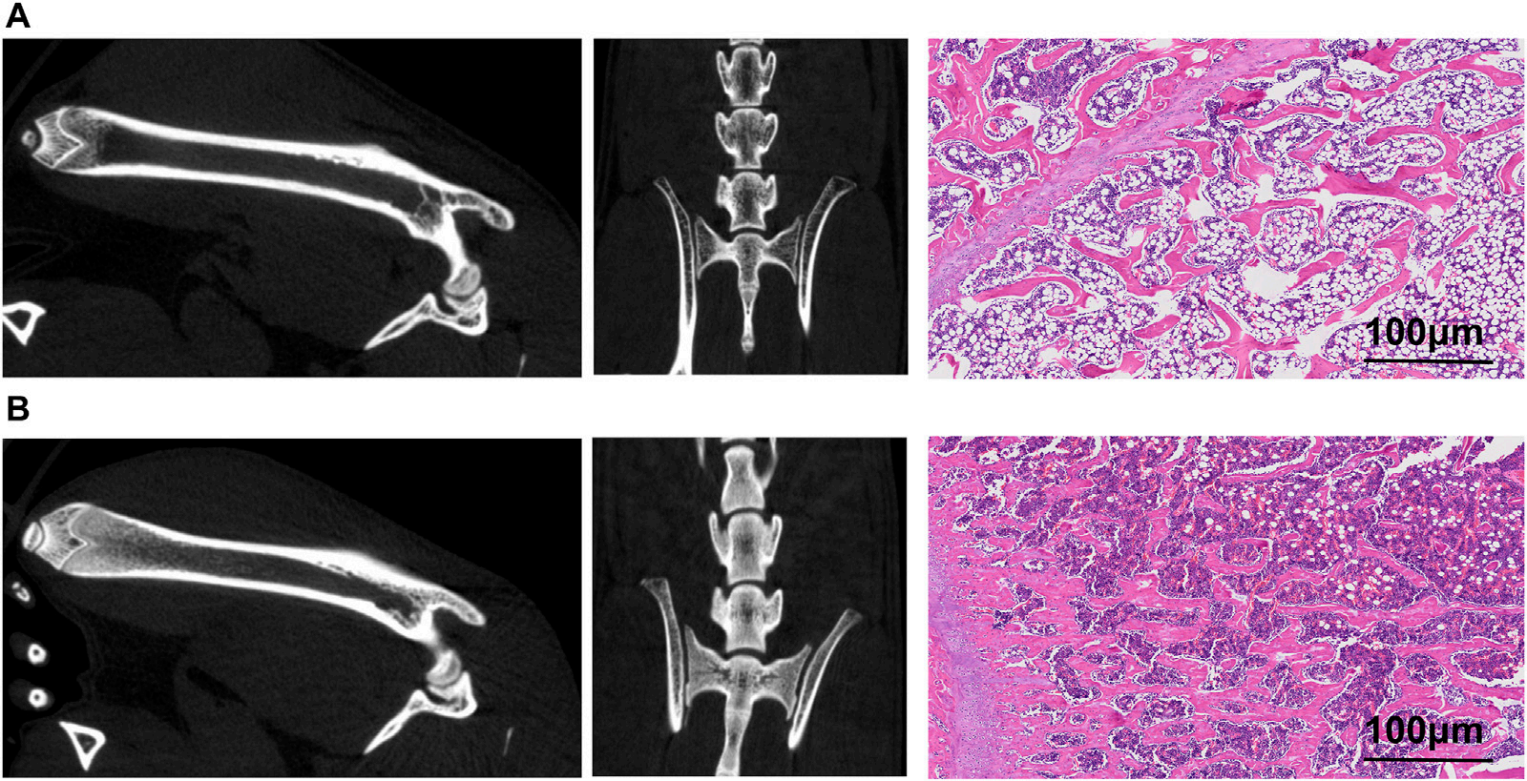
Images of ovariectomy-induced osteoporosis model rat and sham rat. **(A)** Small animal PET/CT images of the right femur and lumbar spine, along with CT scan images and H&E staining pictures of the osteoporosis model rats; **(B)** Small animal PET/CT images of the right femur and lumbar spine, along with CT scan images and H&E staining pictures of the sham rat.

Before evaluating the tracer’s imaging efficacy in the brains, high-resolution CT images from the small-animal PET/CT scans of ^18^F-JNJ-64413739 (Figures 5A, B) were first analyzed to assess the successful formation of the osteoporotic model. These images revealed a significantly lower CT density in the femoral and lumbar medullary cavites of osteoporosis model rats compared to the sham operation group, indicating a reduction of trabeculae and bone density after ovariectomy. Subsequent H&E staining results further confirmed that the trabecular bones in the femur of the osteoporotic model rats were sparse, with more free ends and a destroyed reticular structure. Moreover, the number of vacuolar adipocytes in the bone marrow was significantly increased. In contrast, the sham control group showed intact morphology and structure, with densely arranged and interconnected trabeculae forming a reticular network, and an abundant number of bone marrow cells (Figures 5A, B).

**FIGURE 6:**
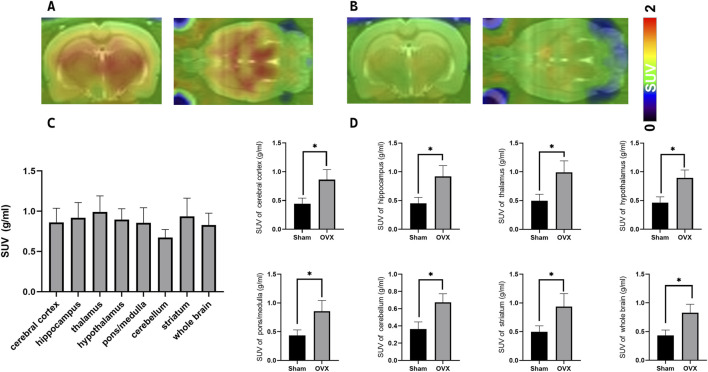
PET/CT Imaging of the P2X7R-targeting probe [^18^F-JNJ64413739 in ovariectomy-induced osteoporosis model rat and sham rat. **(A)** Representative images of osteoporosis model rats at 45 min post-injection; **(B)** Representative images of normal control rats at 40–60 min post-injection; **(C)** Radioactivity uptake in each brain region in different groups; **(D)** Difference of SUV_mean_ values between ovariectomy-induced osteoporosis model rat (n = 3) and sham rat (n = 3),*p < 0.05.

The ^18^F-JNJ-64413739 small-animal PET/CT imaging results revealed significantly higher radioactivity uptake in various brain regions of the osteoporotic model rats compared to the sham group (Figures 6A, B). The highest SUV_mean_ was observed in the thalamus, followed by the striatum, and lowest was in the cerebellum (Figure 6C). Additionally, Figure 6D indicated that the SUV_mean_ values including SUVmean values across the cerebral cortex, hippocampus, thalamus, hypothalamus, pons/medulla, cerebellum, striatum, and whole brain were consistently higher in the OVX group compared with the sham group, further supporting the upregulation of P2X7R expression in the brain of osteoporosis model rats.

**FIGURE 7:**
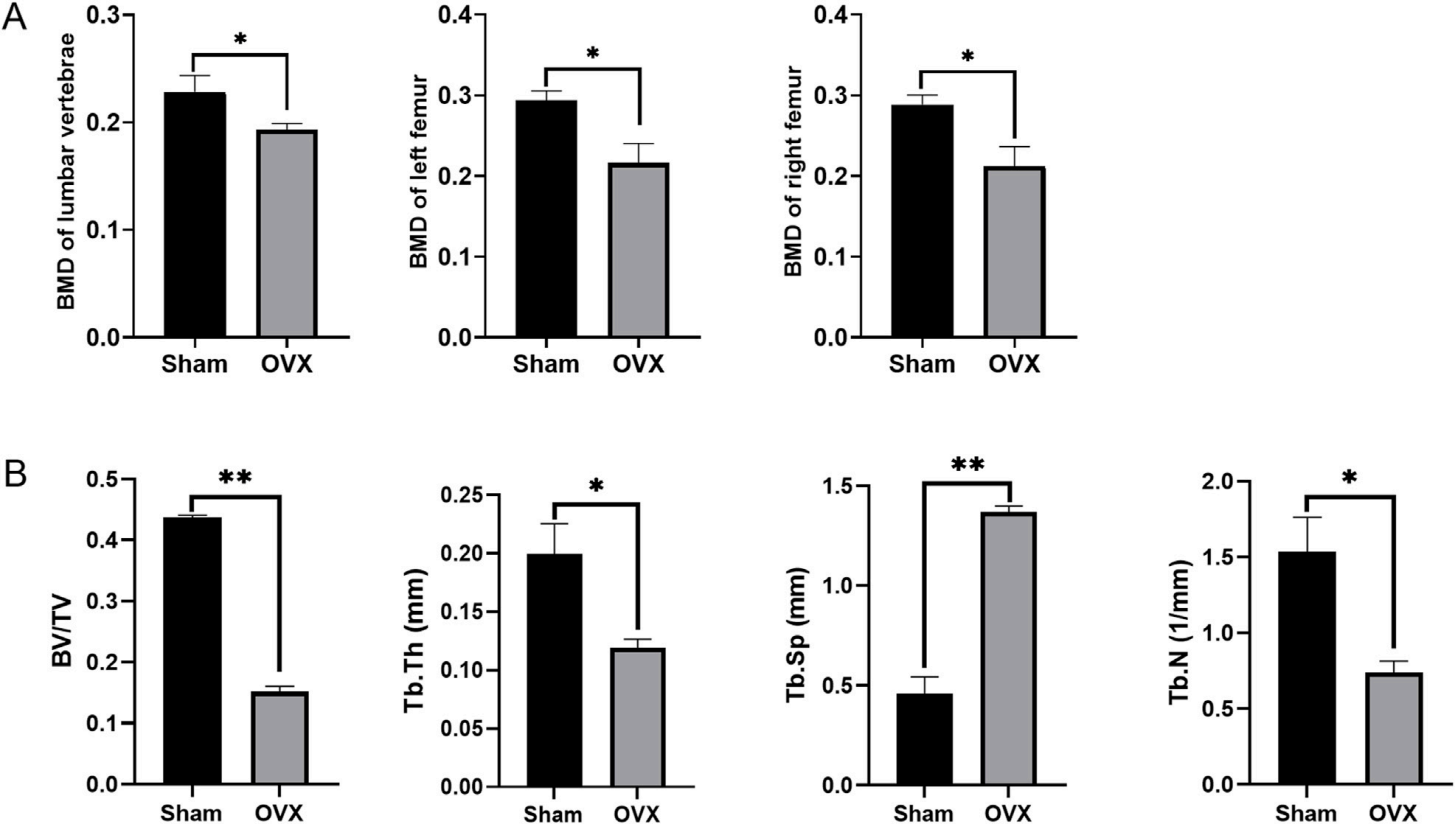
The Bone Mineral Density (BMD) and the micro-CT results. **(A)** The BMD at different positions in different groups; **(B)** The micro-CT results of femurs; *p < 0.05, **p < 0.01, ns: non-significant.

BMD analysis also revealed lower density in the lumbar vertebrae, left femur, and right femur of osteoporotic model rats in the OVX group compared to the sham group (Figure 7A). Micro-CT results showed that in the osteoporotic rats, the BV/TV and Tb.N and Tb.Th were reduced, while Tb. Sp was increased compared to the sham rats (Figure 7B).

## 4 Discussion

^18^F-JNJ-64413739 is a promising PET tracer for P2X7R, but its relatively low synthesis yield (3.1% ± 2.0%) limit its broader application and clinical translation. Our group is dedicated to developing novel PET tracers and advancing their preclinical studies. Considering the chemical structure of ^18^F-JNJ-64413739 as well as the reaction mechanism of nucleophilic aromatic substitution used for radio fluorination, we hypothesized that using a more electron-deficient nitropyridine as the labeling precursor could enhance the labeling efficiency. This motivated us to choose compound 1 for further exploration. Through optimizing the labeling condition, including the screening of different bases and reaction times, we successfully achieved automated synthesis of ^18^F-JNJ-64413739 using the Trasis AllinOne module. The radiochemical purity exceeded 97%, with the highest n. d. c. RCY reached 12%, 3-fold higher than the results obtained with chloropyridine as the precursor using the TRACERlab FX-FN synthesis module (Kolb et al., 2019). However, the robustness of this radiosynthesis remains a challenge, possibly due to the sensitivity of the labeling reaction to environmental humidity. Our team will continue to optimize the synthesis process to enhance the yield stability of the automated production.

Aging is a key risk factors for brain diseases, characterized by a progressive degenerative process often accompanied by chronic low-level inflammation. The ATP-gated P2X7R plays an important role in inflammation and has been implicated in the pathogenesis of several neurodegenerative diseases, including multiple sclerosis, Alzheimer’s disease, Parkinson’s disease, and Huntington’s disease, making it a potential therapeutic target (Zheng et al., 2024). Beyond its involvement in neurological conditions, P2X7R is also involved in age-related disease such as cancer, osteoporosis, diabetes and arthritis (Tao et al., 2013; Kvist et al., 2014; Novak and Solini, 2018; Adinolfi et al., 2018), further positioning it as a therapeutic potential in osteoporosis. Studies reported by Juana Maria Sanz and others suggest that targeting P2X7R could help alleviate inflammation-related brain disorders and other age-associated diseases. In elderly Caucasian populations from high-income countries, hypofunctional P2X7R variants might offer protection against prevalent chronic inflammatory diseases, with hypomorphic P2RX7 alleles potentially under positive selection with age (Sanz et al., 2020). The findings indicate that P2X7R is related to both neurological diseases and osteoporosis, suggesting an important role in bridging these conditions, although research in this area remains limited. This study also conducted ^18^F-JNJ64413739 PET/CT imaging of osteoporotic rats to provide a molecular imaging foundation for further investigations into the role of P2X7R in the brain-bone axis.

This article analyzed the changes of P2X7R in the brains of osteoporosis rats, and found that compared to normal rats, the radioactivity in the thalamus were significantly increased. Previous studies have emphasized the important role of the thalamus in osteoporosis. Yeh CB et al. and Ito A et al. report that calcitonin can partially reverse ovariectomy-induced hypersensitivity in a rat models and alleviate lower back pain in patients with osteoporosis or neuropathic pain by the modulating receptor or channel expression in thalamus (Yeh et al., 2016; Ito and Yoshimura, 2017). Additionally, other literature has shown that oxidative stress can not only contributes to hyperalgesia in OVX mice but also increase osteoporotic pain by affecting the thalamus (Tu et al., 2020). Within the thalamus, the thalamic reticular nucleus (TRN), a thin shell-like cell band located between the outer medullary lamina and the internal capsule, serves as a crucial pathway for almost all axons connecting the thalamus and cortex. Functioning as a gateway between the thalamus and cortex, the TRN facilitates information exchange within the thalamocortical circuit, owing to its unique neural structure and vital anatomical position (Wang et al., 2021). About the striatum, Liping Zhou et al. showed that icariin, a flavonoid phytoestrogen derived from *Herba epimedii*, regulated the transcriptional events of estrogen-responsive genes related to bone remodeling and prevented dopaminergic neurons against OVX-induced changes by rescuing expression of estrogen-regulated tyrosine hydroxylase and dopamine transporter in the striatum (Zhou et al., 2020) showed that considerable P2X7R colocalized with microglia after injection of Abeta (1–42) into rat hippocampus in Alzheimer disease (McLarnon et al., 2006). Literature reports that there is a relationship between hippocampus and bone. Hippocampus participates in the balance of the bone-brain endocrine axis (Patricia da Silva et al., 2023) and significant upregulation of doublecortin protein expression was observed in the hippocampus of both calcium- and vitamin D-treated rats (Lapmanee et al., 2023). Microglia, the immune cells in the central nervous system, are widely distributed throughout these regions. Almost all pathological processes in the brain trigger microglia activation. Therefore, when osteoporosis occurs, the upregulation of P2X7R on microglia in the thalamus becomes significant, though this mechanism has been rarely explored in osteoporosis research.

In summary, this study employed the AllinOne module to complete the automated synthesis of the P2X7R imaging agent ^18^F-JNJ64413739, with non-corrected synthesis yield up to 12%. This synthetic method is simple and reliable, producing a product meets the standards for clinical injection. Imaging results in osteoporotic rats suggest that ^18^F-JNJ64413739 is safe and feasible for use, and the its application holds significant scientific value for exploring the occurrence and progression mechanism of P2X7R-related osteoporosis.

## Data Availability

The raw data supporting the conclusions of this article will be made available by the authors, without undue reservation.
