# Corrigendum: Optimized automated radiosynthesis of ^18^F-JNJ64413739 for purinergic ion channel receptor 7 (P2X7R) imaging in osteoporotic model rats

**DOI:** 10.3389/fphar.2025.1633378

**Published:** 2025-06-04

**Authors:** Yingtong Lu, Yan Cui, Lu Hou, Yuanfang Jiang, Jingjie Shang, Lu Wang, Hao Xu, Weijian Ye, Yang Qiu, Bin Guo

**Affiliations:** ^1^ Department of Nuclear Medicine, The First Affiliated Hospital, Jinan University, Guangzhou, China; ^2^ Traditional Chinese Medicine Department, The First Affiliated Hospital, Jinan University, Guangzhou, China; ^3^ Department of Gynecology, Jiangmen Wuyi Traditional Chinese Medicine Hospital of Jinan University, Jiangmen, Guangdong, China

**Keywords:** P2X7R, osteoporosis, positron emission tomography, isotope labeling, fluorine radioisotopes

In the published article, there was an error in **Affiliation 3**. Instead of “Department of Gynecology, Jiangmen Wuyi Traditional Chinese Medicine Hospital of Jinan University, Guangzhou, Guangdong, China,” it should be “Department of Gynecology, Jiangmen Wuyi Traditional Chinese Medicine Hospital of Jinan University, Jiangmen, Guangdong, China.”

The authors apologize for this error and state that this does not change the scientific conclusions of the article in any way. The original article has been updated.

